# Mechanisms underlying the role of DISC1 in synaptic plasticity

**DOI:** 10.1113/JP274330

**Published:** 2018-07-14

**Authors:** Daniela Tropea, Neil Hardingham, Kirsty Millar, Kevin Fox

**Affiliations:** ^1^ Neurospychiatric Genetics Trinity Center for Health Sciences and Trinity College Institute of Neuroscience (TCIN) Trinity College Dublin Dublin Ireland; ^2^ School of Biosciences Museum Avenue Cardiff University Cardiff UK; ^3^ Centre for Genomic & Experimental Medicine MRC Institute of Genetics & Molecular Medicine Western General Hospital University of Edinburgh Crewe Road Edinburgh UK

## Abstract

Disrupted in schizophrenia 1 (DISC1) is an important hub protein, forming multimeric complexes by self‐association and interacting with a large number of synaptic and cytoskeletal molecules. The synaptic location of DISC1 in the adult brain suggests a role in synaptic plasticity, and indeed, a number of studies have discovered synaptic plasticity impairments in a variety of different DISC1 mutants. This review explores the possibility that DISC1 is an important molecule for organizing proteins involved in synaptic plasticity and examines why mutations in DISC1 impair plasticity. It concentrates on DISC1's role in interacting with synaptic proteins, controlling dendritic structure and cellular trafficking of mRNA, synaptic vesicles and mitochondria. N‐terminal directed mutations appear to impair synaptic plasticity through interactions with phosphodiesterase 4B (PDE4B) and hence protein kinase A (PKA)/GluA1 and PKA/cAMP response element‐binding protein (CREB) signalling pathways, and affect spine structure through interactions with kalirin 7 (Kal‐7) and Rac1. C‐terminal directed mutations also impair plasticity possibly through altered interactions with lissencephaly protein 1 (LIS1) and nuclear distribution protein nudE‐like 1 (NDEL1), thereby affecting developmental processes such as dendritic structure and spine maturation. Many of the same molecules involved in DISC1's cytoskeletal interactions are also involved in intracellular trafficking, raising the possibility that impairments in intracellular trafficking affect cytoskeletal development and vice versa. While the multiplicity of DISC1 protein interactions makes it difficult to pinpoint a single causal signalling pathway, we suggest that the immediate‐term effects of N‐terminal influences on GluA1, Rac1 and CREB, coupled with the developmental effects of C‐terminal influences on trafficking and the cytoskeleton make up the two main branches of DISC1's effect on synaptic plasticity and dendritic spine stability.

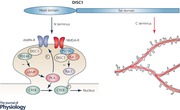

##  

### Introduction

Disrupted in schizophrenia 1 (DISC1) is a protein that when disrupted by a chromosomal t(1;11) translocation (Thomson *et al*. 2016) or mutated (Sachs *et al*. 2005) predisposes the carrier to a number of mental health disorders including schizophrenia, bipolar disorder and recurrent major depression. The synaptic location of DISC1 (Kirkpatrick *et al*. 2006; Carlisle *et al*. 2011), together with recent evidence for its role in cortical synaptic plasticity (Greenhill *et al*. 2015; Tropea *et al*. 2016) raises the question of how DISC1 affects plasticity and whether disruption of plasticity might itself lead to psychiatric symptoms. Indeed, a number of large‐scale genomics studies also implicate copy number variation and mutations in synaptic proteins involved in plasticity as risks factors for psychiatric conditions (Fromer *et al*. 2014; Pocklington *et al*. 2014; Hall *et al*. 2015; Marshall *et al*. 2017). In addition to the pressing need to understand these debilitating disorders, there is a fundamental biological question to answer about the function of DISC1 in the neuron. *In vitro* studies show that DISC1's pleiotropic function occurs through interactions with several proteins (Fig. 1) including phosphodiesterase 4B (PDE4B), glycogen synthase kinase 3 β (GSK3β), kalirin 7 (Kal‐7), fasciculation and elongation protein zeta‐1 (FEZ1), kendrin, lissencephaly protein 1 (LIS1) and nuclear distribution protein nudE‐like 1 (NDEL1)/nuclear distribution protein nudE homolog 1 (NDE1) (Miyoshi *et al*. 2003; Ozeki *et al*. 2003; Brandon *et al*. 2004; Millar *et al*. 2005b; Mao *et al*. 2009; Lipina *et al*. 2012). Which of these pathways are important for plasticity and what processes are altered to disrupt plasticity when DISC1 is mutated?

**Figure 1:**
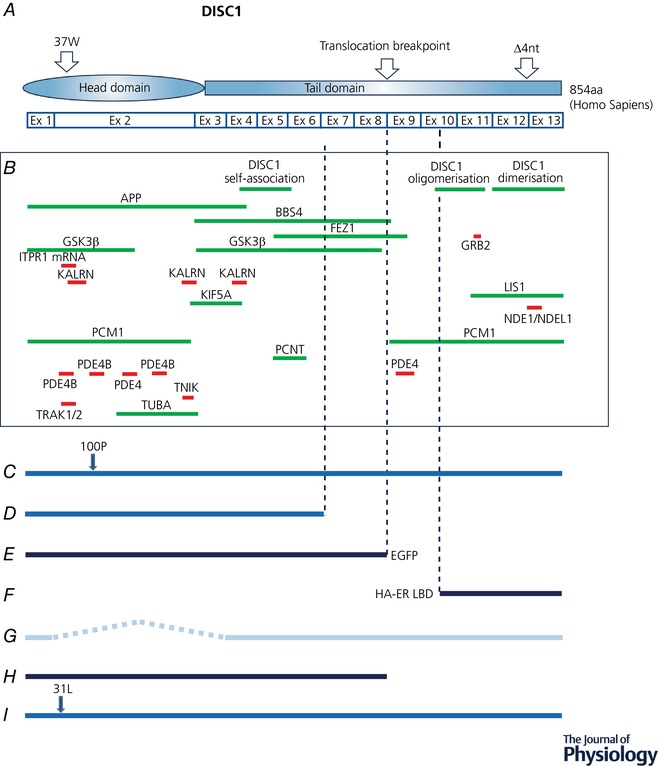
DISC1 protein–protein known interaction sites and DISC1 mouse mutants *A*, schematic representation of DISC1 human protein indicating naturally occurring mutation sites. 37W and the four‐nucleotide deletion in exon 12 are human mutations detected in psychiatric patients (Sachs *et al*. 2005; Song *et al*. 2008; Thomson *et al*. 2013). *B*, self‐association sites and sites required for partner binding/association. Fine‐mapped binding/association sites are indicated in red, and green indicates regions of DISC1 known to contain a site. See Table 1 for references to the original studies reporting the DISC1 protein interactions indicated here. *C*, endogenous mouse DISC1 carrying ethylnitrosourea‐induced point mutation (Clapcote *et al*. 2007). *D*, endogenous mouse DISC1 carrying targeted exon 8 stop codon and intron 8 polyA site plus a natural 25‐nucleotide deletion in exon 6; full‐length DISC1 expression is abolished (Koike *et al*. 2006). *E*, truncated mouse DISC1 with C‐terminal enhanced green fluorescent protein tag, expressed under native DISC1 promoter from transgenic bacterial artificial chromosome (Shen *et al*. 2008). *F*, inducible DISC1 expressed under a CAMKII promoter with N‐terminal mutant oestrogen receptor ligand binding domain fused to HA peptide tag (Li *et al*. 2007). *G*, endogenous mouse DISC1 exon 2/3 deletion; DISC1 expression is abolished (Kuroda *et al*. 2011). *H*, truncated human DISC1 cDNA inducibly expressed under cytomegalovirus (CMV) promoter with N‐terminal Myc peptide tag (Pletnikov *et al*. 2008) *I*, endogenous mouse DISC1 carrying ethylnitrosourea‐induced point mutation (Clapcote *et al*. 2007). *J*, human DISC1 cDNA carrying functional sequence variants, expressed under PrP promoter in rat. Four amino acid sequence changes are indicated; 607F and 704C are common human sequence variants (Trossbach *et al*., 2016). Dark blue lines indicate transgenic DISC1 expression against a background of endogenous expression; mid blue lines indicate modified endogenous expression; pale blue line indicates endogenous gene modification to abolish expression.

DISC1 is known to play a role in early development and has been implicated in neuronal proliferation, cell migration and neurite extension (Brandon *et al*. 2009). Several excellent reviews on DISC1's role in these processes have been published (Brandon and Sawa, 2011; Bradshaw & Porteous, 2012; Wu and Xiao, 2013; Muraki and Tanigaki, 2015) and in this review, we focus instead on synaptic plasticity in the adult as a critical process deficient in animals with mutated DISC1. However, we also consider the possibility that DISC1's action in adulthood is the result of modifications that occur at early phases of postnatal development. In particular, we discuss the hypothesis that through specific protein–protein interactions, DISC1 mediates the impairment of intracellular trafficking, dendritic growth and spine maturation during development, which ultimately lead to alterations in circuit plasticity in adulthood. We begin by considering DISC1's protein–protein interactions, since a crucial element of these plasticity defects is likely to lie in signalling pathways affected by DISC1.

#### Organization of DISC1 protein interactions

Secondary structure analysis using the amino acid sequence of full‐length DISC1 (854 amino acids for human DISC1) predicts that it consists of two major domains, the N‐terminal head domain and the C‐terminal tail (Fig. 1 *A*). The N‐terminal head domain (approximately amino acids 1–350 in human DISC1) exhibits poor evolutionary conservation, with the exception of two highly conserved motifs, the first rich in arginine, and the second rich in serine and phenylalanine (Chubb *et al*. 2008). The head domain is predicted to be largely disordered (Soares *et al*. 2012), implying a structural flexibility that could facilitate binding to multiple targets. On the contrary, the C‐terminal tail domain (approximately amino acids 350–854 in human DISC1) is well conserved across species and predicted to be highly structured, consisting of a series of α‐helices interspersed with at least four regions comprising strong coiled‐coil forming potential (Soares *et al*. 2012).

Tertiary structure analysis throws further light on the nature of DISC1. DISC1 is able to self‐associate to form multimers. Amino acids 403–504 of human DISC1 were the first shown to enable the protein to multimerize (Kamiya *et al*. 2005). This ability was further dissected by generating a series of human DISC1 fragments, with size exclusion chromatography demonstrating that several regions of DISC1, most of which are located within the tail domain, can form multimers or dimers (Leliveld *et al*. 2008; Leliveld *et al*. 2009; Yerabham *et al*. 2017). Size exclusion chromatography also indicates that full‐length DISC1 forms multimers with equilibrium analytical ultracentrifugation, demonstrating that it adopts predominantly dimeric and octameric forms, and that dimers likely act as the building blocks for orderly formation of octamers (Narayanan *et al*. 2011).

DISC1 has a large number of confirmed interactors (see Table 1 and Fig. 1 *B*) and many additional putative binding partners, and it appears to act as a ‘hub’ protein within interaction networks (Camargo *et al*. 2007). It is hypothesized to be a molecular scaffold, assembling multiple proteins into functional units. For some interactions that have been studied in detail, it has been found that there are several contact sites along the length of DISC1. For example, DISC1's interaction with the PDE4 family of cAMP phosphodiesterases was examined using peptide array mapping (Millar *et al*. 2005b; Murdoch *et al*. 2007). This demonstrated the existence of five contact sites for PDE4B (Fig. 1). Of these sites, four are located within the head domain, one encompassing the conserved arginine‐rich region, and another the conserved serine/phenylalanine‐rich region (Murdoch *et al*. 2007). GSK3β and kalirin also appear to contact DISC1 at more than one binding site (Morris *et al*. 2003; Ozeki *et al*. 2003; Brandon *et al*. 2004; Kamiya *et al*. 2006; Mao *et al*. 2009; Hayashi‐Takagi *et al*. 2010), and many DISC1 partner binding sites may overlap (Fig. 1). For example, the arginine‐rich region in the head domain to which PDE4B binds (Murdoch *et al*. 2007) is also essential for DISC1 to complex with trafficking kinesin protein 1/2 (TRAK1/2; Ogawa *et al*. 2014; Norkett *et al*. 2016) and with the 3′‐untranslated region (UTR) of inositol‐1,4,5‐trisphosphate receptor type 1 (ITPR1) mRNA (Tsuboi *et al*. 2015; Fig. 1). This multifunctional arginine‐rich region also acts as a nuclear localization signal (Sawamura *et al*. 2008; Malavasi *et al*. 2012).

**Table 1:** **DISC1 interactors: the DISC1 interactors shown in** **Fig**. 1 **are given alongside their full names and the references first establishing their interactions with DISC1**

Interaction factor	Name	Reference
DISC1	Disrupted in schizophrenia 1	Kamiya *et al*. (2005), Leliveld *et al*. (2009)
APP	Amyloid precursor protein	Young‐Pearse *et al*. (2010)
BBS4	Bardet–Biedl syndrome 4	Kamiya *et al*. (2008)
FEZ1	Fasciculation and elongation protein zeta‐1	Miyoshi *et al*. (2003)
GRB2	Growth factor receptor‐bound protein 2	Shinoda *et al*. (2007)
GSK3	Glycogen synthase kinase‐3	Mao *et al*. (2009)
ITPR1 mRNA	Inositol 1,4,5‐trisphosphate receptor type 1	Tsuboi *et al*. (2015)
KALRN	Kalirin, RhoGEF kinase	Hayashi‐Takagi *et al*. (2010)
KIF5A	Kinesin family member 5A	Taya *et al*. (2007)
LIS1	Lissencephaly 1	Brandon *et al*. (2004)
NDE1/NDEL1	nudE neurodevelopment protein like 1 or NDE like 1	Burdick *et al*. (2008), Ozeki *et al*. (2003)
PCM1	Pericentriolar material 1	Kamiya *et al*. (2008)
PCNT	Pericentrin	Miyoshi *et al*. (2004)
PDE4/PDE4B	Phosphodiesterase 4/phosphodiesterase 4B	Millar *et al*. (2005b)
TNIK	TRAF2 (TNF receptor‐associated factor 2) and NCK‐interacting kinase	Wang *et al*. (2011)
TRAK1/2	Trafficking kinesin protein 1/2	Ogawa *et al*. (2014), Norkett *et al*. (2016)

While it is possible that DISC1 binds only one interactor at any given time (at sites for multiple protein binding), it is also conceivable that the process of multimerization enables simultaneous binding of partners that utilize shared sequences on DISC1. Indeed, the structural information available to date suggests that the tail domain is involved in self‐association, while the head domain is essentially free and could therefore potentially interact with up to eight proteins at the same binding sites within an octameric structure. This scenario is further complicated by data indicating that some binding partners may only interact with a specific type of DISC1 multimer. This could be the case for NDEL1, which contacts a site within the tail domain (Soares *et al*. 2012) and has been reported to preferentially bind DISC1 octamers and oligomers, rather than dimers or higher order multimers formed from recombinant fragments (Leliveld *et al*. 2008; Leliveld *et al*. 2009). However, another study using full‐length DISC1 found that NDEL1 binding is not dependent upon the type of DISC1 oligomer formed (Narayanan *et al*. 2011).

In addition to the orderly formation of multimers, DISC1 has a propensity to aggregate, particularly when overexpressed. Under these conditions, a substantial proportion of DISC1 becomes detergent‐insoluble (Millar *et al*. 2005a; Leliveld *et al*. 2008, 2009; Zhou *et al*. 2010; Atkin *et al*. 2012; Eykelenboom *et al*. 2012) and forms aggresomes (Atkin *et al*. 2012). This may be relevant to psychiatry because detergent‐insoluble DISC1 has been detected in human brain tissue, and at increased levels in postmortem samples from patients diagnosed with schizophrenia or mood disorders (Leliveld *et al*. 2008). Moreover, there is evidence that DISC1 aggregation and insolubility is regulated by dopamine (Trossbach *et al*. 2016), at least in cell culture. Aggregated DISC1 does not interact with NDEL1 (Leliveld *et al*. 2008), indicating that this potentially pathological mechanism could disrupt DISC1 function at many levels, including DISC1 mislocalization, and lead to the loss of critical protein–protein interactions.

Having considered the basic organization of DISC1 interactions, we now consider how they influence synaptic function.

### Synaptic plasticity

Synaptic plasticity is the ability of the synapse to change its transmission characteristics, either to increase its ability to depolarize the neuron by long‐term potentiation (LTP) or to decrease its depolarizing effect by long term depression (LTD). Synaptic plasticity is an important process for synaptic development, as synapses are initially weak when they are first formed, but is also critical in the adult animal for learning, memory and sensory adaptation (Bliss & Collingridge, 1993). Functional changes in synapses are closely linked to structural and molecular rearrangements, which, as characterized in this review, are often deficient in animals with DISC1 mutations.

DISC1 is localized in dendritic spines and in the adult is particularly enriched in the postsynaptic density (PSD) (Kirkpatrick *et al*. 2006; Hayashi‐Takagi *et al*. 2010; Carlisle *et al*. 2011). Curiously, synaptic transmission *per se* appears to be largely intact in most DISC1 mutants (Cui *et al*. 2016), although in some cases it may adopt a less mature form than normal (Greenhill *et al*. 2015). Instead, the main effect of DISC1 on synaptic function appears to be its effect on synaptic plasticity. Synaptic plasticity is often studied in animal models of schizophrenia by measuring LTP, and it is therefore highly pertinent that LTP has been found to be deficient in schizophrenia patients (Frantseva *et al*. 2008). In the following sections, we consider the impact of DISC1 mutations on synaptic plasticity, distinguishing between mutations affecting the N‐terminal or C‐terminal domains of DISC1.

#### N‐terminal effects

A number of proteins interact with the N‐terminal domain of DISC1 including PDE4B, Kal‐7 and GSK3β (Table 1 and Fig. 1 *B*). DISC1 interacts with PDE4B at four sites within the N‐terminal domain and one in the C‐terminal tail (Murdoch *et al*. 2007; Fig. 1). PDE4B works to decyclize cAMP and, as a consequence, it can antagonize protein kinase A (PKA)‐mediated effects including cAMP response element‐binding protein (CREB) activation and phosphorylation of the AMPA receptor subunit GluA1, both of which are important for plasticity.

Ethylnitrosourea‐generated mutant L100P mice have a point mutation at amino acid 100 of the DISC protein, which lies in a PDE4B binding region (Table 2 and Fig. 1 *C*). They have normal basic synaptic transmission and normal expression levels of synaptic proteins, but show major deficits in glutamate‐induced Ca^2+^ elevation. Importantly, L100P mice have impaired hippocampal LTP in slices derived from adult mice (Cui *et al*. 2016) (Fig. 2 *H*).

**Table 2:** **DISC1 mutants: the original and follow‐on studies using the DISC1 mutants depicted in** **Fig**. 1 **are cross‐referenced, together with a description of the mutation being tested**

Mutation	Original report	Further studies using the mutant	Nature of mutation and studied as	Fig. 1 panel
L100P	Clapcote *et al*. (2007)	Lee *et al*. (2011a,*b*), Cui *et al*. (2016), Tropea *et al*. (2016)	Ethylnitrosourea‐induced point mutation (heterozygotes and homozygotes)	*C*
DISC1 Tm1Kara	Koike *et al*. (2006)	Kvajo *et al*. (2008, 2011), Lepagnol‐Bestel *et al*. (2013)	Artificial stop codon and polyadenylation signal at exon 8, combined with deletion within exon 6 (heterozygotes and homozygotes)	*D*
truncated DISC1	Shen *et al*. (2008)	Booth *et al*. (2014)	Transgenic	*E*
DISC1cc	Li *et al*. (2007)	Greenhill *et al*. (2015)	Transgenic	*F*
DISC1 Δ2/3	Kuroda *et al*. (2011)	Tsuboi *et al*. (2015)	knockout (homozygous null)	*G*
hDISC1	Pletnikov *et al*. (2008)		Transgenic	*H*
Q31L	Clapcote *et al*. (2007)		Point mutation (heterozygotes and homozygotes)	*I*

**Figure 2:**
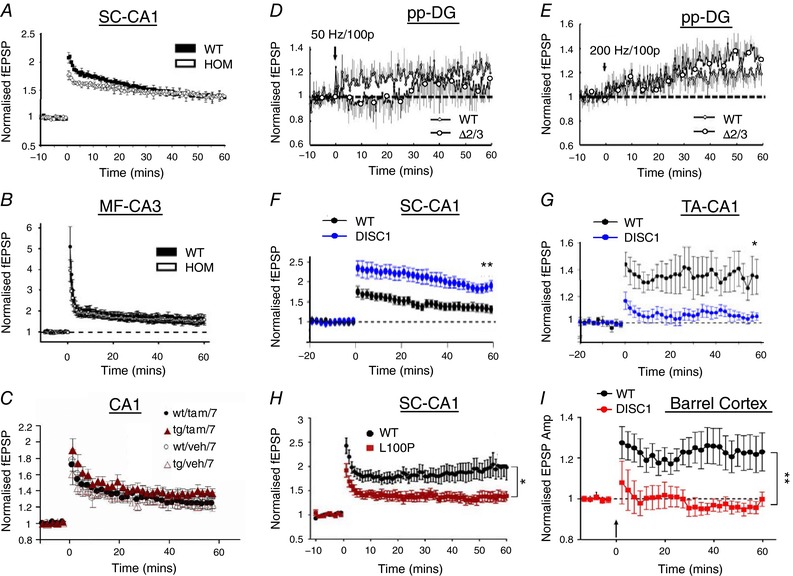
Effects of DISC1 mutations on LTP *A*, an early component of LTP is affected in the hippocampal CA1 (Schaffer collateral input) but LTP is still present (Kvajo *et al*., 2008). *B*, LTP is unaffected in the mossy fibre input to CA3 (Kvajo *et al*. 2011). *C*, LTP is unaffected in CA1 (Li *et al*. 2007). *D* and *E*, the early component of LTP is absent and the later component of LTP attenuated in the perforant pathway input to the dentate gyrus when induced using a 50 Hz tetanus (*D*), but more similar to wild‐types when induced using a 200 Hz tetanus (*E*) (Kuroda *et al*. 2011). *F* and *G*, LTP is enhanced in the Schaffer collateral pathway (*F*) but absent in the temperoammonic pathway of the hippocampus (*G*) (both, Booth *et al*. 2014). *H*, LTP is strongly reduced in the Schaffer collateral pathway (Cui *et al*. 2016). *I*, LTP is absent in the layer 4 to layer 2/3 pathway in the barrel cortex (Greenhill *et al*. 2015).

The impaired plasticity of L100P mice is also present in the neocortex *in vivo* (Tropea *et al*. 2016). Cortical activity and visual map organization are normal, but cortical ocular dominance plasticity is impaired in L100P mice (Tropea *et al*. 2016). Studies have shown that ocular dominance plasticity in the mouse visual cortex is due to a tumour necrosis factor α (TNFα)‐dependent homeostatic mechanism related to synaptic scaling (Kaneko *et al*. 2008; Ranson *et al*. 2012). Blockade of the TNFα mechanism during the critical period of development leads to a loss of potentiation of the open eye response but leaves LTP intact (Stellwagen & Malenka, 2006; Kaneko *et al*. 2008). The mechanism underlying this effect is not known, but phosphorylated CREB and TNFα levels are directly correlated (Datusalia & Sharma, 2014; Miao *et al*. 2015), and so the reduction in phosphorylated CREB by the L100P mutation may down‐regulate levels of TNFα, thereby reducing the homeostatic component of ocular dominance plasticity. Taken together with Cui *et al*’s findings, this implies that the L100P mutation impairs both LTP and homeostatic plasticity, suggesting that it acts at a basic mechanistic level that is common to both types of plasticity (Cui *et al*. 2016). Homeostatic up‐regulation of synaptic strength and LTP both involve AMPA receptor insertion, which could therefore provide a common link. However, current understanding suggests the kinase signalling pathways for LTP and homeostatic plasticity are different; while PKA influences phosphorylation of GluA1 at the S845 site to control GluA1 insertion in the membrane (Lee *et al*. 2000; Kopec *et al*. 2007; Hardingham *et al*. 2008), phosphoinositide‐3‐kinase (PI3K) controls GluA1 insertion through the TNFα mechanism (Stellwagen *et al*. 2005). Nevertheless, AMPA receptor insertion and GluA1 are important both for homeostatic up‐regulation processes (Stellwagen & Malenka, 2006; Goel *et al*. 2011) and ocular dominance plasticity (Ranson *et al*. 2013).

DISC1 interactions with PDE4B also influence plasticity through CREB's effect on gene expression. Monocular deprivation normally produces a reduction in phosphoCREB levels in the visual cortex. However, monocular deprivation fails to reduce phosphoCREB in homozygous L100P mutant mice (measured as a change in phosphoCREB immunostaining) (Tropea *et al*. 2016). Similarly, in neuronal culture, chemically induced LTP (cLTP) increases phosphoCREB levels in wild‐types (WT) but not in the L100P mutants (Tropea *et al*. 2016). Furthermore, whereas cLTP induces an increase in the expression of synaptic proteins in WTs (specifically synapsin and PSD‐95), it fails to generate significant changes in L100P‐derived cortical cultures (Tropea *et al*. 2016). These studies suggest that DISC1 may influence synaptogenesis and/or maturation of synapses via CREB signalling, which is defective in the L100P mutant.

The importance of the PDE4B–DISC1 interaction for the modulation of LTP is further emphasized by experiments on homozygous PDE4B mutant mice, where PDE4B was manipulated so that the affinity for cAMP was either increased or decreased (McGirr *et al*. 2016). Neurons in CA1 did not show LTP after high frequency stimulation in mice where the affinity between PDE4B and cAMP was increased (PDE4B+). PDE4B can influence two stages of LTP; by affecting PKA activation, PDE4B can affect the early phases of LTP (Lee *et al*. 2000; Kopec *et al*. 2007; Hardingham *et al*. 2008) and by affecting CREB activation, PDE4B can affect late phase LTP (Barco *et al*. 2002).

Kal‐7 is another important synaptic DISC1 interactor and binds DISC1 at three sites, two of which are part of the N‐terminal head domain (41–100 and 321–355) and one of which is in the C‐terminal tail domain (376–410) (see Fig. 1). Deletion of amino acids 350–394 was found to strongly affect DISC1–Kal‐7 interactions (Hayashi‐Takagi *et al*. 2010). Interactions between DISC1 and Kal‐7 are important for driving the NMDA‐mediated activation of Ras‐related C3 botulinum toxin substrate 1 (Rac1). Rac1 plays an important role in spine enlargement and shrinkage and, as discussed later, this is one of the mechanisms by which DISC1 can affect spine morphology. In cortical cell cultures, blockade of DISC1 has a biphasic effect on spine size with respect to time. Within 24 h of DISC1 inactivation, spines increase in size and excitatory postsynaptic currents (EPSCs) increase in frequency. In contrast, prolonged down‐regulation of DISC1 over 6 days causes a shrinkage of spines and a reduction in EPSCs (Hayashi‐Takagi *et al*. 2010). One note of caution, however, is that because of the limited lifetime of neuronal dissociated cultures, synapses may not reach the equivalent age at which effects are observed *in vivo*, though they may still give insights into the time course of DISC1 action at the cellular level. The peak of DISC1 expression in mice is P35 (Schurov *et al*. 2004), which is later than most conventional culture methods allow. Nevertheless, it can be seen from the above studies that the N‐terminal head of DISC1 plays an important role in shaping spine structure and plasticity through interactions with Kal‐7 and PDE4B.

#### C‐terminal effects

Experiments have also been performed on mice missing the C‐terminal portion of DISC1. These mutations give insight into the function of the DISC1 C‐terminal tail. In 2006, Koike and colleagues inserted a stop codon into DISC1 exon 8, followed by a polyadenylation signal, and at the same time noted that the mouse strain they had used, 129SvEv, carries a natural deletion in DISC1 exon 6 (Koike *et al*. 2006) (Fig. 1 *D*). This mutation results in an overall diminished expression of DISC1 protein, and very weak expression of a C‐terminally truncated protein corresponding to the size predicted by introduction of a premature stop codon due to the exon 6 deletion (Kvajo *et al*. 2008). Kvajo and colleagues transferred the modified mutated DISC1 allele into a C57BL/6 background and used this mouse model to study hippocampal circuitry. In these mice, they found that LTP can still be elicited in hippocampal CA1 (Fig. 2 *A*), although the initial magnitude of LTP is lower than in WT (Kvajo *et al*. 2008). In a follow‐up study, the authors generated a mouse strain where in addition to the DISC1 mutation, a specific population of neurons expressed green fluorescent protein (GFP) under the Thy1 promoter (Kvajo *et al*. 2011). They found that the axonal projections in the DISC1 mutants were disrupted, particularly those of the granule cells in the dentate gyrus (Kvajo *et al*. 2011), which project to CA3. In CA3, ultramicroscopic examination showed an unaltered number of synaptic vesicles but their size was reduced in mutants. Consistent with this finding, facilitation decreased more rapidly in homozygous mice as the frequency of stimulation was reduced. However, the mice again showed no deficit in LTP in this pathway (Fig. 2 *B*).

During the same period, another DISC1 mouse model was generated (Shen *et al*. 2008). This model was generated by overexpressing two copies of DISC1 exons 1–8 via an artificial chromosome (Fig. 1 *E*). The authors showed many anatomical and morphological alterations in these mice that mimic the neuropsychiatric phenotype, some of which are consistent with reduced neurogenesis and cell migration, such as thinning of cortical layers II/III, and others that are consistent with effects on neurite outgrowth. Booth and colleagues studied electrophysiological properties of this mouse in the hippocampal Schaffer collateral (SC) and temporo‐ammonic (TA) pathway inputs to CA1 (Booth *et al*. 2014). Basal synaptic transmission in mutant mice was relatively normal except that, studying the SC pathway, DISC1 slices were more variable and epileptogenic. Furthermore, the SC pathway produced higher levels of LTP in DISC1 mutants than in WTs (Fig. 2 *F*). In contrast, the TA pathway exhibited a profound reduction in LTP in DISC1‐mutant slices (Fig. 2 *G*). In both cases, theta burst stimulation (TBS) was used to induce LTP. The differences evident between TA and SC pathways in this study show that the same DISC1 mutation can differentially affect alternative inputs onto the same cells.

Booth *et al*.’s study and Kvajo *et al*.’s studies both used a DISC1 model with a C‐terminal truncation (Fig. 1 *D* and *E*), but observed different effects on LTP. There may be several reasons for the discrepancy; first, despite similarities, the animal models do differ: Booth *et al*. used a model with an over‐expression of the truncated form of DISC1, with maintained expression of the endogenous protein, while Kvajo and colleagues used a model with a loss of full‐length protein and an unstable expression of a C‐terminally truncated protein (Fig. 1 and Table 2). Second, Booth and colleague used TBS to induce LTP, while Kvajo *et al*. used high frequency stimulation. It has been shown that TBS is required to induce both nitric oxide and GluA1 components of LTP in the hippocampus, while high frequency stimulation only induces the GluA1 component (Phillips *et al*. 2008). It is conceivable that only one component of LTP was studied in Kvajo *et al*.’s experiments and the nitric oxide component of LTP in Booth *et al*.’s study is deficient in the TA pathway.

All the studies described so far use mutants where the expression of the mutant DISC1 is effective throughout development. However, Li *et al*. (2007) generated a transgenic mouse expressing the C‐terminal portion of DISC1 (Fig. 1 *F*) in pyramidal neurons of the forebrain where the timing of the disruption can be controlled (Li *et al*. 2007). The mutant protein is expressed constitutively, but continually degraded unless tamoxifen is administered, whereupon the C‐terminal fragment of DISC1 becomes active. Once the tamoxifen is metabolized, degradation of the DISC1 fusion protein resumes. This allows for a fast onset and offset of free mutant protein availability (6–48 h with a single tamoxifen injection), which then acts as a dominant negative for endogenous DISC1 by binding to the C‐terminal domain protein binding partners of DISC1, including LIS and NDEL1 (Li *et al*. 2007) and probably also interfering with orderly multimerization of DISC1 (Fig. 1 *A*). The authors disrupted DISC1 function during development or in adulthood, then ran a series of behavioural tests to identify the age at which interference of DISC1 signalling caused most functional disruption. They found that disruption of DISC1 signalling at P7 was most effective at impairing working memory and dendritic complexity in the dentate gyrus, both of which are related to human schizophrenia pathology. However, there was no effect of DISC1 disruption in adulthood, implying a developmental requirement for DISC1.

Li *et al*. found that LTP in the SC pathway in this heterozygous mutant was not reduced by induction of mutant DISC1 at P7, in common with Booth *et al*. (Fig. 2 *C*), but did not test the temporoammonic pathway, which has since been shown to exhibit C‐terminal‐related impairments in LTP (Booth *et al*. 2014).

Further studies on neocortical plasticity have thrown more light on these findings. Greenhill *et al*. (2015) studied the same mouse as Li *et al*. (2007) and found that normal DISC1 function during development is required for adult expression of LTP and experience dependent plasticity in layers 2/3 of the somatosensory cortex (Greenhill *et al*. 2015). Similar to Li and colleagues’ findings, the dominant negative DISC1 fragment did not affect plasticity when activated in the adult (Greenhill *et al*. 2015). However, a single tamoxifen injection at P7 produced a deficit in adult cortical plasticity measured months later (Fig. 2 *I*). Tamoxifen injections to disrupt DISC1 function at P11–13 also impaired adult plasticity, but to a lesser degree than at P7 (Greenhill *et al*. 2015). These findings show that DISC1 is required during a developmentally critical period in order for adult plasticity to develop in the cortex. Once again, spike timing plasticity was used to induce LTP, which affects both GluA1 and NO‐dependent aspects of plasticity (Hardingham & Fox, 2006). This early critical period coincides with the formation of synapses and dendritic branching patterns on the affected neurons (Greenhill *et al*. 2015), which may explain why plasticity is affected in some pathways and not others (Li *et al*. 2007), given that different neuronal pathways develop at different ages. The exact nature of the plasticity affected by early DISC1 dysfunction is quite specific in that it affects adult LTP and experience‐dependent potentiation, but only has a limited effect on LTD and de‐depression (Greenhill *et al*. 2015).

#### Hippocampal *versus* cortical effects

A number of studies have been performed on LTP in the hippocampus using a variety of DISC1 mutants. As shown in Fig. 2 *A*, *B* and *C*, three studies have found little or no effect of DISC1 mutation on LTP in the SC pathway (Li *et al*. 2007; Kvajo *et al*. 2008, 2011) and one actually showed an increase in LTP (Booth *et al*. 2014), possibly due to reduced inhibition in these mutants (Fig. 2 *F*). In these cases the C‐terminal domain of DISC1 was truncated for investigation. The only case where a partial reduction was seen in the level of LTP in the SC pathway was due to an N‐terminal DISC1 mutation (Fig. 2 *H*) (Cui *et al*. 2016).

Plasticity has been studied in other hippocampal pathways and found either to be abolished, as with the TA pathway, or the threshold for plasticity induction affected, as with the perforant path input to the dentate gyrus (Kuroda *et al*. 2011). In the latter case, an effective knockout of both the N‐ and C‐terminal domains of DISC1 was produced by deletion of exons 2 and 3 (Fig. 1 *G*). While it was still possible to trigger LTP using a frequency generally used to investigate LTP (50–100 Hz), LTP was reduced but not eliminated in DISC1 mutant mice (Fig. 2 *D*), while at 200 Hz LTP was greater in the DISC1 mutants than in WTs (Fig. 2 *E*).

These partial and pathway‐specific effects in the hippocampus are for the most part in contrast to the far starker effects of DISC1 mutations on plasticity in the neocortex. Greenhill *et al*. (2015) saw a complete abolition of LTP in layer 2/3 neurons (Fig. 2 *I*) and a loss of whisker deprivation induced experience‐dependent potentiation with a C‐terminally directed DISC1 manipulation, while Tropea *et al*. (2016) saw a clear abolition of ocular dominance plasticity in the visual cortex and a lack of cLTP in cortical cell cultures with an N‐terminally directed DISC1 mutation. Similarly, studies on spine morphology and structural plasticity in the cortex have consistently demonstrated effects of DISC1 mutations (Hayashi‐Takagi *et al*. 2010). It remains to be determined whether these differences are due to intrinsic properties of the neocortex and hippocampus, or whether some of the differences arise due to the different DISC1 mutations used in each study. It is also important to note that ocular dominance plasticity involves LTD and homeostatic plasticity, which are generally not investigated in the hippocampus of DISC1 mutants (LTP is normally measured). One of the major differences between the neocortex and hippocampus is the longevity and stability of synapses. Synapses can last for a period of several months in the neocortex, while most hippocampal synapses turn over within a period of a month (Yang *et al*. 2009a; Attardo *et al*. 2015). If DISC1 does have a different role in the hippocampus and cortex, it could indicate a role for DISC1 in structural plasticity and the long‐term stability of synapses, particularly given the demonstrated role of DISC1 in cytoskeletal interactions and intracellular trafficking, as will be discussed in the following sections.

### Development and control of neuronal morphology

Schizophrenic patients show a deficit in spine density in deep layer 3 cortical pyramidal neurons in the prefrontal cortex (Glantz & Lewis, 2000), and a number of mental disorders have been linked to deficits in dendritic spines (Penzes *et al*. 2011). Dendritic spines contain the postsynaptic compartment and receive the majority of the excitatory synaptic input in the brain and thus changes in dendritic spine morphology are closely linked to synaptic plasticity (Kasai *et al*. 2010). The link between DISC1 and mental disorders has thus prompted research into the effects of DISC1 at the structural level

An initial study on human frontal and parietal cortex showed that a pool of DISC1 is located at the synapse (Kirkpatrick *et al*. 2006) with some 40% of synapses, particularly excitatory synapses, labelled for DISC1. A similar localization of DISC1 at the PSD was shown both in rat cortical and hippocampal cell cultures (Hayashi‐Takagi *et al*. 2010; Wang *et al*. 2011). However, as many studies have shown, the effects of DISC1 are not only imposed at the synaptic level (Fig. 3 *A*). The DISC1 interactome points towards multiple functions of DISC1 during development: effects on neuronal proliferation, migration, neurite outgrowth and the formation and maintenance of synapses (Brandon, 2007; Camargo *et al*. 2007). Whilst changes in spine morphology are most closely linked to synaptic plasticity, it is apparent that any change in neuronal morphology, from incorrect neuronal location to retarded or reduced neurite outgrowth, could lead to incorrect connections being formed between neurons and synaptic plasticity subsequently being affected in the mature brain. In this sense, plasticity could be impaired by malformation of a neuronal circuit. Alternatively, by affecting the dendritic composition during development, DISC1 could permanently affect the properties of the spines that constantly form and retract on the dendrites and thereby affect their ability to undergo synaptic plasticity. Consideration of these hypotheses requires a discussion of DISC1's cytoskeletal interactions.

**Figure 3:**
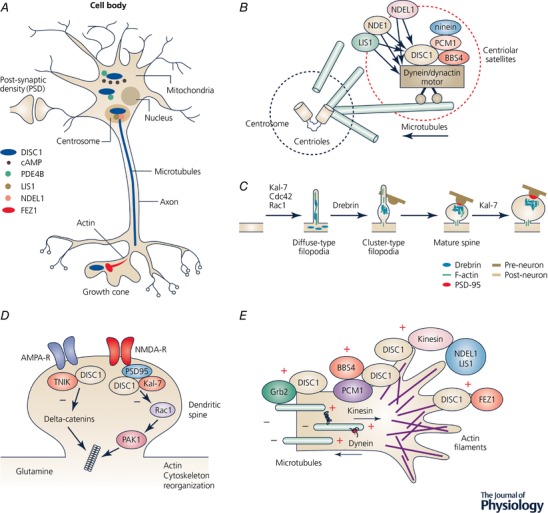
Cellular locations and protein interactions of DISC1 *A*, a schematic representation of a neuronal cell body and dendritic processes, axon‐aligned microtubules and actin‐rich growth cones. DISC1 complexes are found at the centrosome (cream) and mitochondria (black) in the cell body and at the growth cones (invagination at tip of axon). DISC1 interacts with PDE4B at mitochondria and in the cytosol and also localizes to the centrosome where it complexes with NDEL1 and LIS1. DISC1 is also found at the postsynaptic density (expanded in figure) and also colocalizes with FEZ1 at growth cones (also expanded in figure). *B*, DISC1 at the centrosome. DISC1 is known to bind to a number of proteins localized to the centrosome including NDEL1, LIS1, PCM1 and Bardet–Biedl syndrome proteins (BBSs). DISC1 plays a role in anchoring these molecules in association with the dynein motor complex and the centrosome, regulating microtubule organization. *C*, schematic representation of spine formation. During synaptogenesis, dendrites are covered with many diffuse‐type filopodia in which drebrin is diffusely distributed. After an axon terminal makes contact with a filopodium, drebrin clusters with F‐actin at the postsynaptic site and forms a cluster‐type filopodium. PSD‐95 cluster formation follows drebrin–actin cluster formation. The drebrin–actin complex tethers the postsynaptic machinery and is crucial to the maturation of the dendritic spines. Molecules implicated in each of the developmental processes are drawn above the adjoining arrows. Kal‐7, Cdc42 and Rac1 are all implicated in filopodium formation, drebrin in the conversion of filopodia to spines and Kal‐7 is also implicated in spine growth. *D*, DISC1 at the synapse. Multiple lines of evidence show that DISC1 is a component of the postsynaptic density (PSD) of excitatory synapses and regulates their form and function. The effects of DISC1 are mediated through a range of protein interaction partners. Two of these interactors that have been studied considerably are PSD proteins Kal‐7 and TNIK, which both regulate the actin cytoskeleton, most probably via separate pathways. *E*, organization of the axonal growth cone. Microtubules distributed along the axonal shaft extend into the growth cone. The growth cone is enriched with F‐actin bundles that form filopodia or lamellipodia. Cytoplasmic dynein moves along the microtubules towards the minus end, while kinesin moves along the microtubules towards the plus end. Various DISC1 interactors have been implicated in the activation of kinesin, including FEZ1, Grb2, PCM1 and the NDE1/NDEL‐1‐LIS1 complex.

#### Effects on dendrites

Multiple laboratories have shown that DISC1 interacts with proteins that bind to microtubules and associated complexes (Fig. 3 *B*); these proteins include NDE1, NDEL1 and LIS1 (Morris *et al*. 2003; Ozeki *et al*. 2003; Miyoshi *et al*. 2004; Brandon *et al*. 2005; Bradshaw *et al*. 2009; Wang and Brandon, 2011). The role of DISC1 disruption in attenuating neurite outgrowth is well established (Miyoshi *et al*. 2003; Ozeki *et al*. 2003; Pletnikov *et al*. 2008; Shen *et al*. 2008), as is the involvement of NDEL1 and LIS1 in this process, (Kamiya *et al*. 2006; Taya *et al*. 2007; Shim *et al*. 2008). As discussed in more detail in intracellular trafficking (see below), DISC1 is a component of the microtubule‐associated dynein motor complex (Fig. 3 *E*). Disruption of DISC1 early in development has been shown to affect microtubule dynamics and produce a disorganized microtubular network, subsequently impairing neurite outgrowth (Kamiya *et al*. 2005). In these experiments a C‐terminally truncated form of DISC1, which functions as a dominant negative protein, was used to block accumulation of DISC1, NDEL1 and their dynein‐associated proteins at the centrosome (Fig. 3 *B*). This disruption caused a marked retardation in both neuronal migration and neurite outgrowth in the developing cortex (Kamiya *et al*. 2005). The *Disc1*‐L100P mutant mouse has an N‐terminal DISC1 mutation (Fig. 1 *C*) that also produces a change in dendrite and spine organization. Homozygous L100P mice exhibit deficits in both dendrite length and spine density in frontal cortex neurons (Lee *et al*. 2011b). A key mechanism in this effect was shown to be the loss of the interaction between DISC1 and GSK3β, as inactivation of GSK3α rescue spine density in L100P mutant mice but does not rescue spine density (Lee *et al*. 2011b).

Abnormalities in dendritic complexity in C‐terminally truncated DISC1 mutant mice have also been found, with dentate gyrus granule cells from homozygous DISC1^Tm1Kara^ mice showing reduced dendritic length (Kvajo *et al*. 2008, 2011); dendritic length was also reduced in the frontal cortex but not in CA1 hippocampus (Lee *et al*. 2011a). There is evidence that effects of DISC1 mutations are sometimes cell type specific; cultured hippocampal neurons exhibited deficits in both axonal and dendritic length and branching and also at the spine level, but in cultured cortical neurons, reductions in spine density were observed in both heterozygous and homozygous mutant mice (Lepagnol‐Bestel *et al*. 2013). DISC1 has also been demonstrated to regulate neurite outgrowth by controlling cell–cell adhesion and overexpression of DISC1 increased the expression of N‐cadherin and β‐1‐integrin protein as well as enhancing neurite outgrowth (Hattori *et al*. 2010).

Three further studies have examined the role of DISC1 in controlling dendritic morphology at particular stages of development. Pletnikov *et al*. (2008) used the Tet‐off system to express a C‐terminally truncated form of DISC1 (Fig. 1 *H*). This led to decreased neurite outgrowth in primary cortical neurons and reduced levels of LIS1 and SNAP‐25 in forebrain areas (Pletnikov *et al*. 2008). Li *et al*. used a mouse with an inducible and transient (6–48 h) disruption of DISC1 signalling (see ‘Synaptic plasticity’ for details) (Li *et al*. 2007). They found that a single period of disruption at P7 produced chronic reductions in dendritic complexity in the adult hippocampus in transgenic heterozygous mice. Using the same mouse with inducible disruption of DISC1 signalling at P7, Greenhill *et al*. showed that dendritic growth was significantly attenuated in layer 2/3 cortical neurons at both P11 and P14, but returned to normal levels in adulthood, presumably due to the transient expression of the dominant negative protein (Greenhill *et al*. 2015).

#### Effects on dendritic spines

Given the initial observation that schizophrenic patients exhibited a reduced spine density and the link between DISC1 and neurological disorders, it is pertinent that one of the most consistent observations made so far in many DISC1 mutants is a reduction in spine density. The Q31L (Fig. 1 *I*) and L100P (Fig. 1 *C*) mutant mice, which both have mutations in the PDE4B binding sites, show reduced spine density on pyramidal neurons in layers III and V of the frontal cortex and CA1 neurons of the hippocampus in homozygous mutants (Lee *et al*. 2011a). While the DISC1 mutation in this mouse does not occur in human patients, it nevertheless demonstrates the role of DISC1 in spine regulation. The homozygous DISC1^TM1Kara^, carrying a targeted exon 8 stop codon and creating a mouse with truncated DISC1 and no full length DISC1 expression (Fig. 1 *D*), also shows a decrease in synaptic spines in the dentate gyrus and in layer 2/3 prefrontal cortex (or PFC) neurons, but not in layer 5 neurons (Kvajo *et al*. 2008; Juan *et al*. 2014; Crabtree *et al*. 2017). Cultured hippocampal neurons from these mice showed reductions in mushroom and stubby spines but not reductions in filopodia, whilst cultured cortical neurons showed reductions in all classes of spines in both heterozygous and homozygous mutant mice (Lepagnol‐Bestel *et al*. 2013). Long‐term suppression of DISC1 has also been shown to produce changes at the spine level in cultured cortical neurons, with reductions in spine density and effects on spine morphology, specifically reductions in spine size and spine length (Hayashi‐Takagi *et al*. 2010).

In concert with this finding, a disruption of DISC1 signalling for 6–48 h (dominant negative, Fig. 1 *F*) starting at P7 produced almost immediate deficits in spine density just 24 h later at P8. Furthermore, even though the DISC1 disruption only lasted 48 h, the deficit in spine density lasted into adulthood and was accompanied by lasting deficits in synaptic plasticity (see previous section) (Greenhill *et al*. 2015). At P7, layer 2/3 neurons show immature morphology with short dendrites and limited dendritic branching (Greenhill *et al*. 2015). Only primary neurites are developed at this time and higher order dendrites are in the midst of developing (Greenhill *et al*. 2015). The reductions in spine density caused by DISC1 disruption only occurred on second and third order dendrites, i.e. those dendrites developing at the time that DISC1 function was impaired. Among the spines that did develop following DISC1 disruption, a higher proportion than normal were thin spines and a lower proportion were mushroom spines (Greenhill *et al*. 2015). Since spine morphology is representative of maturation state, with thin spines often associated with an early stage of synapse stabilization and maturation, while mushroom‐type spines are associated with stabilized synapses important for synaptic transmission (Tropea *et al*. 2010), one can infer that DISC1 has an effect both on synaptic density and on spine maturation.

How does DISC1 affect spine density and maturation? Regulation of the actin cytoskeleton is one of the cellular processes enriched in the DISC1 interactome (Camargo *et al*. 2007). The actin cytoskeleton is critical in regulating growth cone motility (Fig. 3 *E*), spine formation (Fig. 3 *C*) and underlying synaptic plasticity (Ramakers 2002; Sekino *et al*. 2007; Cingolani & Goda, 2008). An increasing number of intracellular signalling pathways have been shown to regulate the actin cytoskeleton and to be critical for spine structure (Calabrese *et al*. 2006). The RhoGTPases Cdc42 and Rac1, which regulate the outgrowth of the actin cytoskeleton, are involved in the formation of filopodia (Kozma *et al*. 1997) and are also implicated in the maintenance of dendritic spines and thus in the regulation of spine density (Nakayama *et al*. 2000). Rho proteins regulate cell adhesion through proteins linked to the actin cytoskeleton, such as cadherins and integrins, which are present at synapses and are also involved in synaptic plasticity (Ramakers, 2002). As we have already noted (see ‘Synaptic plasticity’), DISC1 binds to Kal‐7 and prevents access of Kal‐7 to Rac1 (Fig. 3 *D*), controlling the duration and intensity of Rac1 activation in response to NMDA activation, when the DISC1 anchor to Kal‐7 is weakened, leading to subsequent spine enlargement (Hayashi‐Takagi *et al*. 2010). Kal‐7 also increases spine formation when overexpressed (Hayashi‐Takagi *et al*. 2010) and there is a strong correlation found between spine density and Duo (the human version of Kal‐7) in schizophrenic patients (Hill *et al*. 2006).

DISC1 also interacts with another kinase, Traf2 and Nck interacting kinase (TNIK), in the dendritic spine (Fig. 1 *B*), and inhibits the kinase activity of TNIK (Wang *et al*. 2011). TNIK is specifically expressed in the brain and highly enriched in the PSD, while inhibition of TNIK leads to loss of PSD95, surface GluA1 and reductions in both mEPSC frequency and mEPSC amplitude (Hussain *et al*. 2010; Wang *et al*. 2011; Burette *et al*. 2015). Furthermore, knockdown of TNIK signalling through RNAi leads to reductions in hippocampal dendritic branching and spine density (Hussain *et al*. 2010). Recent studies suggest these effects may be mediated by δ‐catenin family proteins, part of the classic cadherin adhesion complexes at synapses required for synaptogenesis, spine growth and synaptic plasticity (Wang *et al*. 2016).

Haploinsufficiency in LIS1, one of the major binding partners at the C‐terminal domain of DISC1 (Fig. 1 *B*), has also been shown to reduce spine density both in hippocampal CA1 and barrel cortex and also filopodial length in CA1, with a lower spine turnover and elimination *in vivo*, while down‐regulation of RhoA rescued spine motility. Therefore, this is likely to be a pathway involved in DISC1's effects on spines (Sudarov *et al*. 2013). GSK3α is known to be a specific modulator of spine density in L100P mice, because its genetic inactivation rescues spine density in L100P/GSK3α mutants, while all the other morphological alterations remain unchanged (Lee *et al*. 2011b). Interestingly, Dixdc1 also affects dendritic spines and interacts with DISC1 to control GSK3 and Wnt signalling (Martin *et al*. 2018), though unlike the other interactors mentioned in this section, it is not yet known to be located synaptically. In conclusion, there is overwhelming evidence that DISC1 is important for dendritic spine structure and function. Interestingly, DISC1 can also influence development of the presynaptic side of the synapse, as will be discussed in the following section.

#### Effects on axons

In addition to its established role in dendritic development, DISC1 is also involved in axonal development, most notably in granule cells in the dentate gyrus projecting to their targets in CA3 (Kvajo *et al*. 2011) but also in cultured hippocampal neurons (Lepgagnol‐Bestel *et al*. 2013). FEZ1 and pericentriolar material (PCM1) are two microtubule interacting proteins that bind to DISC1 (Fig. 1 *B*) and are linked to axonal development. Centrosomal PCM1 is required for correct development of axonal morphology and is recruited co‐operatively by interacting proteins DISC1 and Bardet–Biedl syndrome 4 (BBS4) (Kamiya *et al*. 2008; de Anda *et al*. 2010), while alterations in axonal targeting may well involve abnormal elevations in cAMP, as correction of cAMP levels was found to be a means of reversing the deficits (Kvajo *et al*. 2011).

FEZ1 has been identified as an important DISC1 interacting partner that participates in neurite outgrowth and co‐localizes with DISC1 at axonal growth cones (Fig. 1 *B*), whilst interactions are associated with F‐actin (Fig. 3 *E*) (Fujita *et al*. 2007). FEZ1 is known to be involved in the activation of the kinesin‐1 motor protein and in the transport of mitochondria (Blasius *et al*. 2007; Fujita *et al*. 2007) (see ‘Mitochondrial trafficking’ below). The expression of FEZ1 is again developmentally controlled and regulated synchronously with DISC1 (Honda *et al*. 2004). FEZ1, and by implication DISC1, are thus thought to function in axon growth and guidance, possibly involving cytoskeletal remodelling at the axonal growth cone (Miyoshi *et al*. 2003). Consistent with this, up‐regulation of the DISC1/FEZ1 interaction enhances the extension of neurites while silencing of DISC1 inhibits neurite production (Miyoshi *et al*. 2003; Kamiya *et al*. 2005). DISC1 also plays a role in axon elongation through an interaction with growth factor receptor‐bound protein 2 (GRB2), resulting in activation of extracellular signal‐regulated kinase (ERK) (Fig. 3 *E*) (Shinoda *et al*. 2007), or through kinesin and the NDEL1–LIS1 complex in axonal growth cones (Shinoda *et al*. 2007; Taya *et al*. 2007), while knockdown of DISC1, kinesin1, NDEL1 or LIS1 inhibited axon elongation (Taya *et al*. 2007).

Thus, while it is clear that DISC1 perturbation affects various aspects of neurogenesis, neural migration, neurite growth and both spinogenesis and spine maintenance, questions remain about how these processes are controlled. At a superficial level, it is possible to imagine that many of the processes involved in cytoskeletal organization during migration and neurite outgrowth are also involved in the maintenance and plasticity of dendrites and dendritic spines, as discussed above. Given the role of DISC1 in synaptic plasticity and spine morphology, particularly in the neocortex, understanding this process is likely to help in understanding spine plasticity and stability as well as the consequences of disrupting it. One further process that also involves DISC1, which may affect both the development and maintenance of neuronal structure, is neuronal trafficking, and this topic is discussed in the next section.

### Neuronal intracellular trafficking

Due to their highly elongated morphology, neurons are reliant upon efficient microtubule‐based transport of various cargoes between soma and synapses, which can be a considerable distance in some neurons. This process utilizes the molecular motors kinesin and dynein, which transport cargo in the anterograde (away from the soma) or retrograde (towards the soma) directions, respectively, along the polarized microtubules in axons (Fig. 3 *E*). As discussed below, both kinesin and dynein are DISC1 interactors. Dendrites contain microtubule bundles of mixed polarity and thus cargo transport is more complex in this neuronal compartment (Franker & Hoogenraad, 2013). In axons and dendrites, many cargoes undergo bidirectional transport, often exhibiting saltatory movements in both directions, but with overall movement in a single direction eventually achieved (Franker & Hoogenraad, 2013). The mechanism underlying this pattern of movement is unknown, but cargoes appear to be simultaneously complexed with both dynein and kinesin, and it has been proposed that a ‘tug‐of‐war’ occurs between the opposing motors, with one ultimately predominating to determine the overall direction of travel (Bryantseva & Zhapparova, 2012). This hypothesis is, however, complicated by the observation that disruption of the activity of either kinesin or dynein affects movement in both directions (Franker & Hoogenraad, 2013).

A general role is emerging for DISC1 in microtubule‐based cargo transport because it complexes with both kinesin and dynein. DISC1 affinity chromatography using rat brain lysates demonstrated that DISC1 interacts or associates with kinesin family member (KIF) 1B, KIF5A, KIF5B, KIF5C and kinesin light chains 1 and 2 (KLC1 and KLC2) (Shinoda *et al*. 2007; Taya *et al*. 2007; Tsuboi *et al*. 2015). Dynein intermediate chain (DIC) was also found to complex with DISC1 by co‐immunoprecipitation from PC12 cells (Kamiya *et al*. 2005) and by DISC1 affinity chromatography (Taya *et al*. 2007). DISC1 is therefore well placed to regulate both kinesin and dynein, possibly co‐ordinately, and/or to connect cargo with these motors to facilitate their anterograde and retrograde trafficking within axons and dendrites.

There are currently few mechanistic clues as to how DISC1 affects kinesin activity and anterograde cargo transport, apart from a possible role in facilitating cargo recruitment to kinesin adaptors (Flores *et al*. 2011), or in kinesin activation through an interaction with FEZ1 (Blasius *et al*. 2007). More is known, however, about its role in dynein regulation. Dynein activity is controlled by the multiprotein dynactin complex with which DISC1 associates (Kamiya *et al*. 2005), and by the DISC1 binding partner LIS1 (Ozeki *et al*. 2003; Brandon *et al*. 2004)(Burdick *et al*. 2008; Bradshaw *et al*. 2009) and its accessory proteins NDE1 and NDEL1 (Cianfrocco *et al*. 2015; Bradshaw & Hayashi, 2017). The DISC1 interactor and cAMP phosphodiesterase PDE4 also interacts with LIS1 (Murdoch *et al*. 2011; Houslay *et al*. 2017), NDE1 (Bradshaw *et al*. 2008) and NDEL1 (Collins *et al*. 2008), and DISC1 and PDE4 control the composition of LIS1–NDE1–NDEL1 complexes via cAMP‐dependent phosphorylation (Collins *et al*. 2008; Bradshaw *et al*. 2011; Murdoch *et al*. 2011). It follows that DISC1 is likely to regulate retrograde cargo transport through interactions with LIS1, NDE1 and NDEL1 (Bradshaw *et al*. 2011; Murdoch *et al*. 2011), and that this mechanism may involve the second messenger cAMP, although the latter has yet to be proven.

Consistent with the above, DISC1 is now known to modulate motility of diverse cargoes in neurons. It is suggested to be involved in endocytosis of amyloid precursor protein (APP) (Shahani *et al*. 2015) and GABA_A_ receptor trafficking (Wei *et al*. 2015), but the best studied DISC1‐modulated cargoes to date are mitochondria, messenger RNAs and synaptic vesicles. In the following sections we will discuss how defective trafficking of these three cargoes is likely to affect synapse function and plasticity.

#### Mitochondrial trafficking

The brain uses approximately 20% of all the energy utilized by the body at rest, and most of this energy is used by neurons (Harris *et al*. 2012). Mitochondria provide the majority of energy utilized by neurons, in the form of ATP (Harris *et al*. 2012). Much of this ATP consumption is due to neurotransmission, for example driving synaptic vesicle cycling in dendrites and powering ion pumps at the post‐synapse (Harris *et al*. 2012). Mitochondria also power all aspects of neuron development. Of particular relevance to this review, mitochondria are abundant at axonal growth cones and are required for dendritic spine remodelling (Li *et al*. 2004). Neurons are therefore extremely sensitive to mitochondrial defects and/or mitochondrial trafficking dysfunction.

DISC1 is directly involved in bidirectional mitochondrial motility through interaction with the adaptor proteins trafficking kinesin‐binding protein (TRAK) 1 and TRAK2 (Schwarz, 2013; Ogawa *et al*. 2014; Norkett *et al*. 2016). Both TRAK1 and TRAK2 also bind mitochondrial Rho GTPase (Ras homolog family member T1/2 (RHOT1/2, also known as MIRO1/2)), which is embedded in the outer mitochondrial membrane (Schwarz, 2013), where they therefore help link mitochondria to the molecular motors that drive their bidirectional transport along neuronal processes. TRAK1 and TRAK2 interact with DISC1 through a highly conserved arginine‐rich motif within the DISC1 head domain that is essential for both TRAK1 and TRAK2 binding (Ogawa *et al*. 2014; Norkett *et al*. 2016) and mitochondrial localization of DISC1 (Ogawa *et al*. 2014), indicating that the TRAK proteins recruit DISC1 to mitochondria.

Time‐lapse imaging of live neurons containing fluorescently labelled mitochondria has demonstrated that DISC1 regulates mitochondrial motility. DISC1 RNA interference and overexpression reduce and increase the proportion of moving mitochondria in axons, respectively (Atkin *et al*. 2012), while DISC1 overexpression also promotes axonal movement in the anterograde direction at the expense of retrograde movement (Ogawa *et al*. 2014). In contrast, a common DISC1 sequence variant, involving a change of leucine to phenylalanine at position 607 (607F, Fig. 1 *A*) that influences brain structure and function (Thomson *et al*. 2013), abolishes the ability of exogenous DISC1 to increase axonal mitochondrial motility (Atkin *et al*. 2012). Moreover, a rare sequence change of arginine to tryptophan at position 37 (37W), found only in four psychiatric patients to date (Song *et al*. 2008; Thomson *et al*. 2014), blocks the ability of exogenous DISC1 to promote anterograde axonal mitochondrial movement (Ogawa *et al*. 2014). Leucine 607 is located within a putative leucine zipper (Thomson *et al*. 2013), while arginine 37 is located within the conserved arginine‐rich motif that is required for DISC1–TRAK interaction (Ogawa *et al*. 2014; Norkett *et al*. 2016). Indeed, the 37W variant increases DISC1–TRAK1 binding and decreases TRAK1–MIRO binding (Ogawa *et al*. 2014), which may contribute to its effect on axonal mitochondrial transport regulation by DISC1. As well as these sequence variants, an aberrant C‐terminally truncated chimeric form of DISC1, which may arise from the t(1;11) translocation, has been investigated in relation to mitochondrial motility. This DISC1 species is strongly targeted to mitochondria and causes extreme mitochondrial dysfunction when artificially overexpressed (Eykelenboom *et al*. 2012). It also specifically inhibits axonal mitochondrial motility when overexpressed (Norkett *et al*. 2016), although it is not clear whether this is a direct effect, or whether it is due to neuronal pathology as a result of mitochondrial dysfunction. In addition to these axonal effects, DISC1 also regulates mitochondrial motility within dendrites (Norkett *et al*. 2016).

The DISC1 interactor NDE1 (Burdick *et al*. 2008; Bradshaw *et al*. 2009) also complexes with TRAK1 (Ogawa *et al*. 2016) and controls mitochondrial motility. As predicted from its role as a dynein activator (Liang *et al*. 2004; Li *et al*. 2005; McKenney *et al*. 2010), NDE1 promotes retrograde axonal mitochondrial transport (Ogawa *et al*. 2016). The NDE1 interactors LIS1 and NDEL1 also regulate axonal mitochondrial motility (Shao *et al*. 2013). Although LIS1 and NDEL1 have not yet been directly linked to the mitochondrial trafficking machinery, since NDE1 is a component, it is probable that LIS1 and NDEL1 are also components of the transportation complex that are required for dynein regulation.

GSK3β is another TRAK1‐associated DISC1 interactor (Mao *et al*. 2011; Ogawa *et al*. 2016). Its role in mitochondrial trafficking has been examined in several studies (Murphy & Millar, 2017) and, while the results are somewhat conflicting, there is broad agreement that GSK3β regulates the number of motile mitochondria, as well as their velocity, primarily in the anterograde direction (Murphy & Millar, 2017). Moreover, the DISC1 607F variant interferes with DISC1/GSK3β binding (Singh *et al*. 2011) and may therefore disrupt DISC1's ability to promote mitochondrial motility via a loss of GSK3β function in the trafficking complex.

Altogether, these observations indicate that DISC1 co‐ordinates directional mitochondrial movement through its binding partners. Another DISC1 interactor, FEZ1 (Miyoshi *et al*. 2003), may also be involved because it is known to specifically regulate mitochondrial trafficking (Butkevich *et al*. 2016), although its role in this process has not yet been examined with respect to its interaction with DISC1.

As well as being transported along neuronal processes, mitochondria must also respond to stop signals at specific sites, where functions such as mitochondrial ATP production can take place. NMDA receptor‐mediated calcium influx is one of the major signals that causes mitochondria to stop, and this occurs via the calcium‐sensing activity of MIRO (Macaskill *et al*. 2009; Wang & Schwarz, 2009). Another mechanism arresting mitochondrial movement involves syntaphilin (SNPH) which has been described as a docking receptor for axonal mitochondria, since it associates with both mitochondria and the microtubules to immobilize mitochondria (Kang *et al*. 2008). DISC1 may associate with SNPH to arrest axonal mitochondrial movement in response to neuronal activation (Park *et al*. 2016). DISC1 and its binding partners therefore regulate multiple aspects of mitochondrial trafficking, and neurons are thus critically dependent upon these proteins for their energy requirements to be met for proper development and synaptic transmission.

#### Dendritic messenger RNA transport

Synaptic activity is partly regulated by localized protein expression, achieved through control of dendritic distribution and translation of mRNAs (Klein *et al*. 2016). At least 2550 mRNAs are known to be present in dendrites, and localization of many of these is dependent upon structural elements within their 3′‐UTR (Klein *et al*. 2016).

A role for DISC1 in dendritic mRNA targeting was revealed by DISC1 affinity chromatography, which demonstrated its association with the RNA binding proteins heterogeneous nuclear ribonucleoproteins (hnRNP), synaptotagmin‐binding cytoplasmic RNA interacting protein (SYNCRIP), hematopoietic zinc finger (HZF), PURα and receptor for activated C kinase 1 (RACK1), as well as LIS1 and KIF5A (Tsuboi *et al*. 2015). Of these proteins, SYNCRIP and HZF are components of RNA granules that transport and regulate translation of mRNA in dendrites (Bannai *et al*. 2004; Iijima *et al*. 2005; Duning *et al*. 2008). Consistent with this, DISC1 was found to co‐localize with markers of RNA granules within axons and dendrites (Tsuboi *et al*. 2015). HZF binds the 3′‐UTR of mRNA encoding the intracellular calcium release channel ITPR1 (Iijima *et al*. 2005), while live neuron time‐lapse imaging demonstrated that DISC1 is co‐transported bidirectionally with fluorescently tagged ITPR1 mRNA 3′‐UTR within dendrites (Tsuboi *et al*. 2015), suggesting a role for DISC1 in dendritic ITPR1 mRNA transport. To examine this possibility further, a mutant mouse was studied in which DISC1 exons 2 and 3 were knocked out (Fig. 1 *G*) (Kuroda *et al*. 2011). Cultured neurons from this mutant mouse, which lacks any full‐length DISC1 expression, exhibit decreased ITPR1 3′‐UTR transport (Tsuboi *et al*. 2015), confirming a role for DISC1 in dendritic ITPR1 mRNA trafficking. RNA binding assays were then used to demonstrate that DISC1 binds directly to the 3′‐UTR of ITPR1 mRNA in an HZF‐dependent manner, and this was found to involve the conserved multifunctional arginine‐rich motif within the head domain of DISC1 (Tsuboi *et al*. 2015) that is also required for mitochondrial localization and trafficking (Ogawa *et al*. 2014).

ITPR1 transcripts encode an inositol 1,4,5,‐trisphosphate (IP_3_) receptor that mediates release of intracellular calcium to control several cellular functions including neurotransmission and synaptic plasticity (Fedorenko *et al*. 2014), indicating a novel route by which DISC1 can control neuronal function. Importantly, however, DISC1 was also found to bind to the 3′‐UTRs from a number of other mRNAs that are trafficked within dendrites. Of the 40 dendritic transcripts tested for DISC1 binding, mRNAs encoding the DISC1 interactor kalirin, which regulates neuronal morphology and plasticity (see ‘Effects on dendritic spines’) (Hayashi‐Takagi *et al*. 2010), and the voltage‐gated potassium channels KCNC1 and KCNC4 were identified as being DISC1‐associated (Tsuboi *et al*. 2015). Transcripts from the *CACNA1C* and *CACNA2D1* genes encoding voltage‐gated calcium channel subunits were also found to be DISC1‐associated. Taking each in turn, Cav1.2 subunits encoded by the *CACNA1C* gene operate postsynaptically at glutamatergic synapses, where they regulate synaptic plasticity through the CREB signalling pathway and synaptic expression of calcium‐permeable AMPA receptors (Moosmang *et al*. 2005), while at GABAergic synapses they regulate short term GABAergic plasticity (Yang *et al*. 2009b). Second, the *CACNA2D1* gene encodes auxiliary α2/δ1 subunits that regulate presynaptic voltage‐gated calcium channel trafficking, plasma membrane expression and biophysical properties, as well as synaptogenesis (Yang *et al*. 2009b).

The mRNA transport mechanism involves KIF5A (Tsuboi *et al*. 2015), and since the RNA granules mediating mRNA trafficking move bidirectionally (Tsuboi *et al*. 2015), dynein, regulated by LIS1, may also participate in RNA granule transport. Moreover, since DISC1‐associated RNA granules were detected in axons as well as dendrites (albeit to a lesser extent) (Tsuboi *et al*. 2015), it is possible that DISC1 also modulates axonal mRNA trafficking, consistent with the postsynaptic location of some of the encoded proteins. This role in mRNA trafficking implicates DISC1 in regulation of the localized expression of a specific set of proteins that regulate neurotransmission and is likely related to effects on late phase LTP in the DISC1 knockout mouse (Fig. 1 *G*, see also ‘Synaptic plasticity’) (Tsuboi *et al*. 2015). Indeed, disruption of DISC1–mRNA binding blocks the maintenance of LTP (Tsuboi *et al*. 2015). Notably, however, the strategy used to disrupt DISC1–mRNA binding, a peptide encompassing the DISC1 arginine‐rich region, is also likely to disrupt the DISC1 interaction with TRAK1/2 and therefore affect mitochondrial trafficking (Fig. 1 *B*). It may also affect the binding of PDE4B and kalirin to DISC1 (Fig. 1 *B*). In conclusion, there is good evidence that DISC1 can affect mRNA trafficking of several factors involved in synaptic structure and function. In the final section on trafficking we consider the evidence regarding the role of DISC1 in trafficking proteins in synaptic vesicles.

#### Synaptic vesicle transport

Synaptic vesicles store various neurotransmitters that are released into the synaptic cleft in response to presynaptic depolarization‐induced calcium ion influx through voltage‐gated calcium channels. Following neurotransmitter release, the synaptic vesicles are endocytosed, recycled and reloaded with neurotransmitter. These processes require large amounts of energy in the form of ATP.

Regulation of synaptic vesicle movement along neuronal processes by DISC1 was first demonstrated by tagging vesicles with a fluorescently labelled synaptic vesicle marker, vesicle‐associated membrane protein 2 (VAMP2), in cultured mouse neurons (Flores *et al*. 2011). DISC1 knockdown by RNA interference was found to cause aberrant vesicle distribution in neuronal processes, and live neuron time‐lapse imaging demonstrated diminished synaptic vesicle movement. Overexpression of a C‐terminally truncated form of DISC1 also decreased synaptic vesicle motility, and intriguingly, this effect was reversed by treatment with lithium, which is in clinical use as a mood stabiliser (Flores *et al*. 2011). This mechanism involves the DISC1 interactor FEZ1 (Miyoshi *et al*. 2003), which binds to and facilitates activation of kinesin (Blasius *et al*. 2007). FEZ1 regulates synaptic vesicle transport and binds to the synaptic vesicle membrane protein synaptotagmin‐1 (SYT1) in *Drosophila* (Gindhart *et al*. 2003; Toda *et al*. 2008). In mammalian cells DISC1 enhances FEZ1–SYT1 interaction, with the enhancing effect blocked by the C‐terminally truncated DISC1 species and rescued by lithium treatment (Flores *et al*. 2011). DISC1 therefore promotes synaptic vesicle movement by linking synaptic vesicles to kinesin through its interactor FEZ1.

The next study to demonstrate involvement of DISC1 in the synaptic vesicle cycle utilized induced pluripotent stem cell (IPSC)‐derived neurons (Wen *et al*. 2014) from a family carrying a 4 bp deletion in DISC1 exon 12 (Sachs *et al*. 2005). This mutation causes a shift of frame and introduces a premature stop codon (Sachs *et al*. 2005) and is likely to affect DISC1 binding to NDE1 and NDEL1, as well as potentially affecting DISC1 binding to LIS1 and the orderly assembly of DISC1 multimers (Fig. 1 *A* and *B*; see also ‘Organization of DISC1 protein interactions’). Several members of this family have a diagnosis of psychiatric illness, and of those examined, two mutation carriers have schizophrenia and one carrier has schizoaffective disorder, while two non‐carriers have major depression and one non‐carrier has a schizotypal personality disorder (Sachs *et al*. 2005). It has been suggested that the DISC1 mutation is linked to mental illness in this family, although co‐segregation of the mutation with psychiatric illness is incomplete. In IPSC‐derived neurons, mutant DISC1 is expressed normally at the transcript level, but the protein expression is substantially reduced (Wen *et al*. 2014). Mutant neurons express lower levels of the synaptic vesicle marker SV2 and, consistent with this, mutant neurons also exhibit a lower frequency of excitatory spontaneous synaptic currents, implying a defect in presynaptic neurotransmitter release (Wen *et al*. 2014). This was further investigated using the dye FM1‐43, which is intensely fluorescent when taken up by synaptic vesicles. Potassium chloride‐induced synaptic vesicle release was quantified using this dye, and fluorescence reduction over time was found to be lower in mutant neurons, confirming a deficit in synaptic vesicle release (Wen *et al*. 2014). The involvement of DISC1 in these effects was confirmed by correcting the DISC1 expression in mutant lines, and introducing the DISC1 mutation into control lines. Expression of a number of synaptic proteins was altered in the mutant neurons, including increased expression of synapsin (SYN) 2 and SYN3 (Wen *et al*. 2014). Upregulation of SYN1 has previously been shown to suppress neurotransmitter release (Hackett *et al*. 1990; Rosahl *et al*. 1993), and therefore it is possible that increased SYN2 and SYN3 expression acts similarly. Expression of the transcription factor myocyte‐specific enhancer factor 2C (MEF2C) was decreased in the mutant neurons (Wen *et al*. 2014); this is a change which has previously been shown to decrease the frequency of spontaneous synaptic currents (Barbosa *et al*. 2008). This DISC1 mutation therefore dysregulates synaptic vesicle release.

The influence of DISC1 on synaptic vesicle cycling has also been examined using pHluorin fused to the synaptic vesicle protein vesicular glutamate transporter 1 (VGLUT1; Tang *et al*. 2016). pHluorin is a pH‐sensitive form of GFP that fluoresces when released from acidic synaptic vesicles, and that is quenched following reuptake during synaptic vesicle recycling. Release of vesicles labelled with VGLUT1–pHluorin was triggered using trains of action potentials in neurons with DISC1 knocked down using RNA interference. DISC1 knockdown revealed a decreased rate and amplitude of vesicle exocytosis and a trend towards slower endocytosis (Tang *et al*. 2016). When this experiment was repeated with neurons cultured from the homozygous mutant mouse lacking DISC1 exons 2 and 3, which lacks full‐length DISC1 expression (Fig. 1 *G*; Kuroda *et al*. 2011), similar effects were observed on vesicle exocytosis (Tang *et al*. 2016). As calcium ion influx through voltage‐gated calcium channels triggers synaptic vesicle release, the presynaptic marker synaptophysin, fused to the calcium indicator GCamp3, was used to examine presynaptic calcium currents, and action potential trains were used to stimulate calcium transients. DISC1 knockdown or knockout of DISC1 exons 2 and 3 produced a reduction in calcium transients (Kuroda *et al*. 2011; Tang *et al*. 2016). In neurons with DISC1 expression knocked down, this effect was reversed by increasing the extracellular calcium concentration, indicating that in wild‐type neurons DISC1 may facilitate presynaptic calcium ion influx (Tang *et al*. 2016). Calcium channel‐specific blockers were then used to demonstrate that the measured calcium transients are largely mediated by N‐type voltage‐gated calcium channels (CAV2.2), although presynaptic CAV2.2 protein expression is not significantly influenced by DISC1 (Tang *et al*. 2016). The authors concluded that DISC1 enhances synaptic vesicle release mediated by N‐type voltage‐gated calcium channels and that this may be due to altered calcium channel function rather than differential expression of channel proteins (Tang *et al*. 2016).

Taken together, these three studies demonstrate that (1) DISC1 promotes synaptic vesicle transport along neuronal processes, which may, in turn, affect the size of the presynaptic vesicle pools; (2) DISC1 promotes the depolarization‐induced presynaptic calcium influx that drives vesicle exocytosis and neurotransmitter release into the synaptic cleft; and (3) DISC1 promotes neurotransmitter release into the synaptic cleft. The latter may therefore be due to combined effects on vesicle trafficking and calcium influx. Moreover, since mitochondrial ATP is believed to drive both the synaptic vesicle cycle and the ion pumps that restore calcium gradients following presynaptic depolarization (Zenisek & Matthews, 2000; Harris *et al*. 2012), it is possible that DISC1's role in mitochondrial trafficking also contributes to its regulation of the presynaptic calcium current and subsequent neurotransmitter release. Deficiencies in any of these processes are likely to alter the amount of neurotransmitter released into the synaptic cleft and will affect the postsynaptic responses.

In summary, DISC1 has been shown to regulate trafficking of three important neuronal cargoes, mitochondria, mRNA and synaptic vesicles, whose co‐ordinated action is required for neurotransmission. It is possible that future work will extend these observations to additional cargoes by extending the preliminary findings on a role for DISC1 in APP (Shahani *et al*. 2015) or GABA_A_ receptor trafficking (Wei *et al*. 2015). In addition, the DISC1‐associated adaptor TRAK1 is involved in endosome to lysosome trafficking (Webber *et al*. 2008) as well as mitochondrial motility, while TRAK2 is required for movement of the voltage‐gated potassium channel Kir2.1 (Grishin *et al*. 2006) and, like DISC1, is implicated in GABA_A_ receptor transport (Stephenson, 2014). Moreover the DISC1 interactor and kinesin adaptor FEZ1 potentially traffics a large number of proteins required for synaptic transmission and neural development (Butkevich *et al*. 2016). DISC1 may therefore have a general role in transportation of cargo along axons and dendrites to facilitate neurotransmission. Available data indicate that the motility defects observed in response to DISC1 overexpression or knockdown, mutation or knockout may be due to deficits in cargo recruitment to the motors, and/or to effects upon the activity of either dynein, kinesin or both motor proteins.

### Conclusions

Irrespective of its role in schizophrenia, studies on DISC1 have revealed this molecule to be of fundamental importance in many neuronal processes including synaptic plasticity, dendritic growth and intracellular trafficking. These three aspects of DISC1 function are likely to be interrelated and in some cases causal. For example, by altering cellular trafficking, DISC1 can affect dendritic growth, axonal growth and synapse formation, which can then subsequently affect circuit formation and plasticity within the system. However, DISC1 function does not just affect plasticity by affecting the development of neuronal circuits. N‐terminal DISC1 mutations affect plasticity in the adult through their signalling interactions with PDE4B and Kal‐7. These studies imply a continuous role for DISC1 in synaptic organization and hence in synaptic plasticity. It is therefore important to note that among the great variety of DISC1 mutations that have been studied, impairment of normal DISC1 function consistently leads to decreases in synapse density.

However, there are still a number of areas that remain to be understood, most notably how synaptic DISC1 is involved in the stability and plasticity of adult synapses. Due to the variety of DISC1 signalling interactions, there are potentially many ways in which this could happen. Does DISC1 act via PDE4B and hence PKA‐related signalling pathways to control AMPA receptor phosphorylation and CREB activation? Similar considerations could be given to TNIK and Kal‐7 pathways. Does DISC1 act via trafficking of important molecules required for synaptic function in the adult such as synaptic vesicles, messenger RNA and mitochondria? Finally, how does DISC1 alter development of the neuron in such a way as to affect synaptic plasticity in the adult? Answers to these questions should lead not only to a greater understanding of normal synaptic function and plasticity, but also to an understanding of the aberrant synaptic function and plasticity encountered in mental health conditions such as schizophrenia.

## Additional information

### Competing interests

None declared.
